# Draft Genome Sequences of *Lactobacillus plantarum* Strain 90sk and *Lactobacillus brevis* Strain 15f: Focusing on Neurotransmitter Genes

**DOI:** 10.1128/genomeA.00261-15

**Published:** 2015-04-16

**Authors:** Roman A. Yunes, Ksenia M. Klimina, Kirill V. Emelyanov, Natalia V. Zakharevich, Elena U. Poluektova, Valery N. Danilenko

**Affiliations:** Vavilov Institute of General Genetics RAS, Moscow, Russia

## Abstract

The genomes of *Lactobacillus plantarum* strain 90sk and *Lactobacillus brevis* strain 15f were isolated from human intestinal microbiota. Both strains synthesize gamma-aminobutyric acid (GABA), the major inhibitory neurotransmitter. Detailed genome analyses will help to understand the role of GABA in the interaction of bacteria with human intestinal cells.

## GENOME ANNOUNCEMENT

The microbiota of the human body is one of the largest communities of microorganisms, and the most important part is the intestinal microbiota. The totality of microorganisms of intestinal microbiota contributes to the digestive process, inhibits the growth of pathogenic organisms, and participates in the formation of innate and acquired immunity and in the functioning of the gut-brain axis (1). Cell-cell signaling of bacteria from the intestinal microbiota with each other and with the cells of macroorganisms is performed with the assistance of signaling molecules and different small messengers, including neurotransmitters (2, 3). Most human genes encoding enzymes involved in the metabolism of neurotransmitters are potentially microbially derived (4).

*Lactobacillus plantarum* and *Lactobacillus brevis*—facultative heterofermentative lactic acid bacteria—can be isolated from different environments (5), and they are also found in the human intestinal microbiota (6). Some strains of these species are used as probiotics (7, 8). *L. plantarum* strain 90sk and *L. brevis* strain 15f were isolated from healthy adults inhabiting central Russia (from a gut biopsy [L. plantarum 90sk] and from feces [L. brevis 15f]). Both strains synthesize and secrete into the culture medium gamma-aminobutyric acid (GABA), the major inhibitory neurotransmitter in the mammalian central nervous system.

The genomic DNA of *L. plantarum* strain 90sk and *L. brevis* strain 15f was isolated with the Sigma GenElute bacterial genomic DNA kit. The genome sequencing of both strains was carried out using a whole-genome shotgun sequencing approach performed on a Roche 454-GS Junior instrument (Roche, Switzerland). *De novo* genome assembly was performed using the GS *De Novo* Assembler version 2.8 (Roche). Gene predictions and annotations were performed with the NCBI Prokaryotic Genome Annotation Pipeline (PGAAP) (http://www.ncbi.nlm.nih.gov/genome/annotation_prok).

The *L. plantarum* 90sk assembly yielded 40 contigs with a combined length of 3,372,871 bp, 25× genome coverage, and 43.50% GC content. The *L. plantarum* 90sk genome contains 2,969 coding sequences (CDSs), 2 rRNA operons, 64 tRNA genes, and 158 pseudogenes; a CRISPR-related system was not found.

The *L. brevis* 15f assembly yielded 99 contigs with a combined length of 2,440,004 bp, 46× genome coverage, and 45.90% GC content. The *L. brevis* 15f genome contains 2,232 CDSs, 6 rRNA operons, 57 tRNA genes, 97 pseudogenes, and one potential CRISPR-related system.

Genes that contribute to the synthesis and transport of GABA (*gadB* and *gadC* for *L. plantarum* 90sk; *gadRCA* and *gadB* for *L. brevis* 15f), and genes of glutamate transport (*glnGHMP*) have been identified in the genomes of the strains. Genes involved in the synthesis of other neuroactive compounds were also fond: four genes of tyramine operon in the genome of *L. brevis* 15f and a gene of linoleic acid isomerase in the genome of *L. plantarum* 90sk. Detailed genome analysis will help to understand the role of GABA and other small messengers in the interaction of bacteria with human intestinal cells.

### Nucleotide sequence accession numbers. 

These whole-genome shotgun projects have been deposited in GenBank under the accession numbers JXAX00000000 (*L. plantarum* 90sk) and JXCD00000000 (*L. brevis* 15f). The versions described in this paper are the first versions.
